# CD133-targeted oncolytic adenovirus demonstrates anti-tumor effect in colorectal cancer

**DOI:** 10.18632/oncotarget.18340

**Published:** 2017-06-02

**Authors:** Mizuho Sato-Dahlman, Yoshiaki Miura, Jing Li Huang, Praveensingh Hajeri, Kari Jacobsen, Julia Davydova, Masato Yamamoto

**Affiliations:** ^1^ Department of Surgery, University of Minnesota, Minneapolis, Minnesota, USA; ^2^ Masonic Cancer Center, University of Minnesota, Minneapolis, Minnesota, USA; ^3^ Stem Cell Institute, University of Minnesota, Minneapolis, Minnesota, USA

**Keywords:** oncolytic adenovirus, targeting vector, colorectal cancer, CD133, cancer stem cells

## Abstract

Oncolytic Adenoviruses (OAds) are one of the most promising anti-cancer agents that can induce cancer specific cell death. Recently, we generated infectivity-selective OAd, and the resultant OAd tumor-specific binding shows strong efficacy and mitigates toxicity. In this study, we applied this strategy based on adenovirus library screening system for generation of CD133-targeted OAd, and examined their oncolytic activity against colorectal cancer (CRC) *in vitro* and *in vivo*. CD133 (Prominin-1) is an important cell surface marker of cancer stem (like) cells (CSCs) in various cancers, including CRC. Elimination of CSCs has a high likelihood to improve CRC treatment because CSCs population in the tumor contributes to recurrence, metastases, chemotherapy resistance, and poor survival. The OAd with CD133-targeting motif (AdML-TYML) selectively infected CD133^+^ cultured cells and lysed them efficiently. Treatment with AdML-TYML prior to tumor inoculation inhibited the establishment of tumor of CD133^+^ CRC cell lines in nude mice. AdML-TYML also showed strong antitumor effect after intratumoral injections in already established CD133^+^ CRC subcutaneous xenografts. Our results indicate that CD133-targeted OAd selectively infected CD133^+^ CRC, and exhibited anti-tumorigenicity and therapeutic effect in established tumors. This novel infectivity selective virus could be a potent tool for the prevention of metastases and relapses in CRC.

## INTRODUCTION

Colorectal cancer (CRC) is the third most common cancer in the world and the second leading cause of cancer death in the US [1]. The treatment of colorectal cancer has made great progress lately, however, about 50% of patients relapse after treatment [2]. Tumor recurrence and metastasis are two critical survival-influencing factors of CRC. Recent researches suggest that cancer stem cells (CSCs) are responsible for CRC recurrence and metastasis, and also associated with tumor growth, chemo- resistance [3, 4]. Not only in CRC, CSCs have been isolated from a number of tumor types, including brain [5], breast [6], ovarian [7], head and neck [8], pancreas [9] and liver [10]. In this sense, elimination of CSCs has a high potential to improve CRC treatment, and development of therapeutics targeting CSC makes a good clinical sense in a variety of cancers.

In colorectal cancer, several cell surface markers have been identified as cancer stem cell markers. CD133 (also known as human Prominin-1) is a five-transmembrane molecule which has been identified as a cancer stem cell marker in various tumor entities [11], including colon cancer [12, 13]. CD133^+^ cells have shown increased tumorigenicity, self-renewal pathway signaling, and metastasis, as compared with CD133^−^ cells in several cancers [14–16]. Indeed, subpopulations of CD133^+^ colon cancer cells have been demonstrated to exhibit high tumorigenic potential in *in vitro* and *in vivo* [17–19]. Furthermore, Galizia *et al.* demonstrated that CD133 expression is directly associated with number of nodal metastases and subsequent tumor progression [20]. It is therefore reasonable to develop novel therapeutic strategies to attack colorectal CSCs by targeting CD133.

Oncolytic virotherapy is very promising therapeutic approach employing cytocidal function of viruses in order to kill cancer cells. Among them, adenovirus (Ad) is frequently used backbone viruses for the development of oncolytic agents, due to its advantage of high *in vivo* transduction efficiency [21, 22]. However, many cancer cells, including gastrointestinal cancers, lack expression of the primary Ad receptor (Coxackie Adenovirus Receptor, CAR) [23, 24] limiting the infectivity of Ads. If specific binding capability to the cancer specific molecule on the surface of the target cells could be engineered into oncolytic Ads (OAds), the resultant viruses would be capable of overcoming this barrier, and fully realizing the potential of OAd *via* selective delivery to cancer cells through binding of targeted surface markers. For this purpose, we recently established a high-throughput screening method of the fiber-modified adenovirus library, which allows for the isolation of transductionally-targeted adenovirus that selectively binds to cell surface molecule [25].

In this study, we provide a proof of concept for the generating cancer stem-cell specific oncolytic adenovirus and its enhanced therapeutic effect. To this end, we focused on the CD133 as a target molecule for colon cancer stem cells and showed that a potent CD133-targeting OAd derived from the high-throughput Ad library screening has selective cytocidal effect. The resultant CD133-targeting OAd was effectively killed cancer stem cell-like colon cancer cells in a variety of *in vitro* assays and showed anti-tumor effect in xenograft models as well. Our novel therapeutic modality of targeting CD133^+^ cells will have a potential to prevent metastases and relapses of CRC.

## RESULTS

### Isolation of CD133-targeted oncolytic adenovirus by fiber-modified Ad library screening

**Figure 1:**
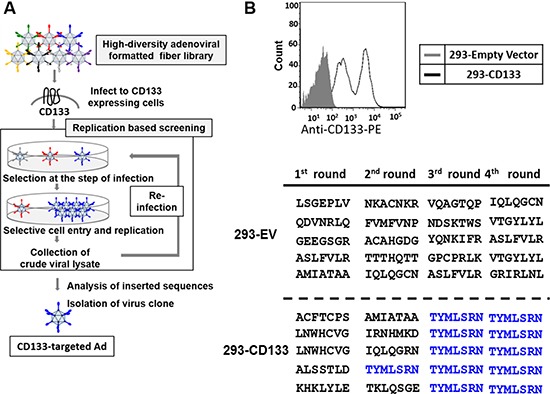
Isolation of CD133-targeted oncolytic adenovirus by high throughput screening of OAd with binding-motif library (**A**) High throughput screening of the OAd library including a targeting ligand library (5 × 10^9^ diversity) was performed with CD133-expressing 293 cells (293-CD133). After 5–7 days, the OAd showing amplification in the 293-CD133 cells were recovered and subjected to the next round of infection. The targeting motif sequences of the viruses were assessed after each round. (**B**) During the screening with CD133 overexpressing cells (293-CD133), the DNA sequences of the AB-loop region of the Ad library were amplified by PCR and cloned into a plasmid. The AB-loop sequences converged to a single clone (TYMLSRN).

A high-diversity adenovirus-formatted library consisting of seven random amino acids in the AB-loop of the fiber-knob region was generated with the recently reported library system [25]. In order to isolate the CD133-targeting OAd, high-throughput screening of the Ad library was performed with target-overexpressing cells based on selective binding and replication (Figure 1A). 293 cells overexpressing CD133 (293-CD133) were infected with the Ad library, and the viral DNA was extracted from crude viral lysate in each round. Subsequently, the region corresponding to the AB-loop of Ad was sequenced after cloning into the plasmid. The “TYMLSRN” motif started to appear in the second round, and became dominant in the third and subsequent rounds (Figure 1B). Such convergence was not observed the negative control cells (293-EV, 293 cells transduced with empty vector). To avoid the effect of potential mutation(s) in other regions of the OAd, the synthetic sequence coding “TYMLSRN” motif was cloned into the AB-loop of the fiber region of the wild type backbone, and the reconstructed CD133-targeted OAd with (AdML-TYML) was used in later experiments.

### Specificity of CD133-targeted oncolytic adenoviruses

**Figure 2:**
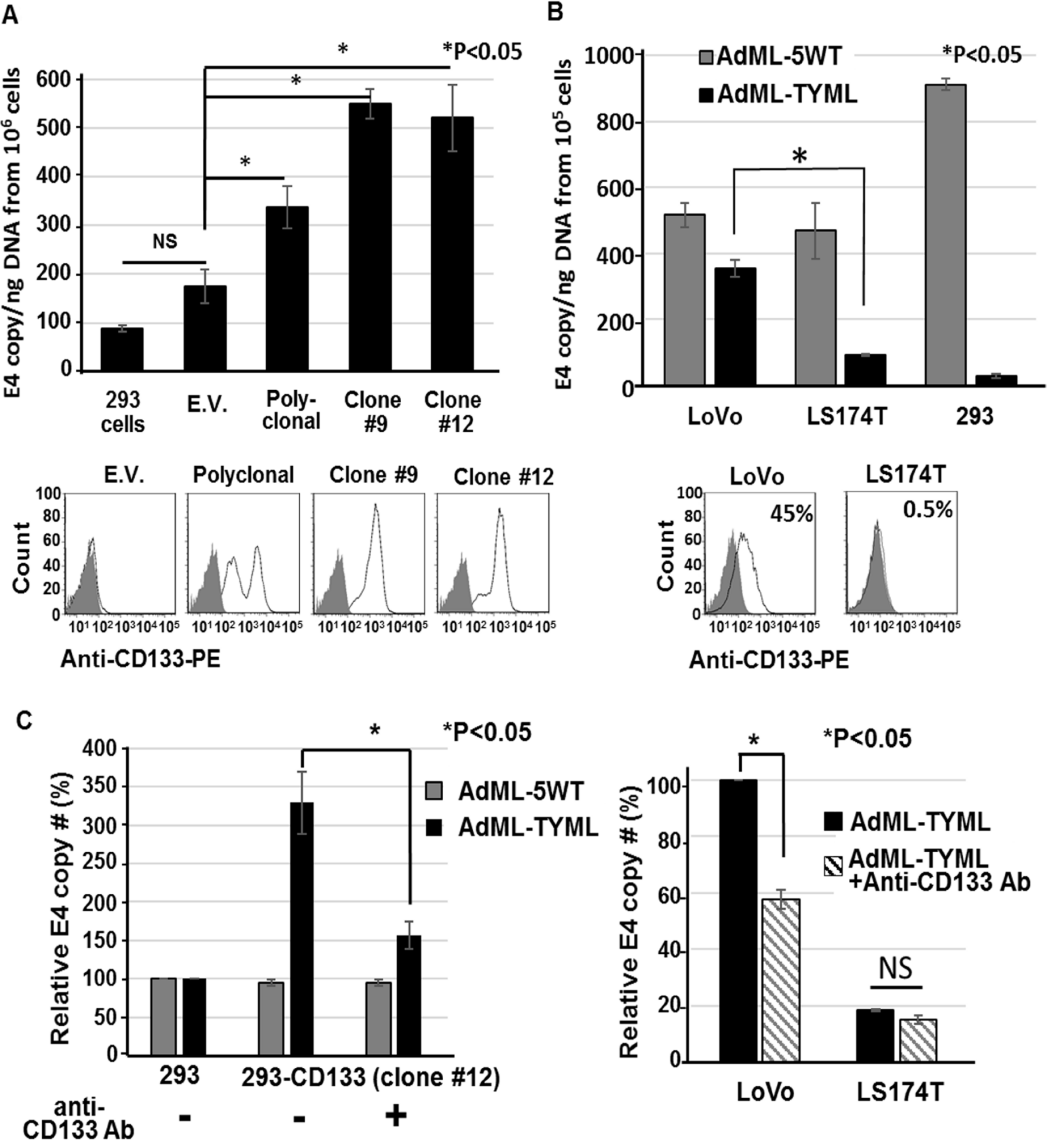
Selective binding of CD133-targeted oncolytic adenoviruses (**A**) Binding specificity of AdML-TYML was analyzed with 293 cells expressing different level of CD133: 293, 293-CD133 (polyclonal mix), isolated clones #9 and #12 (CD133 high expressing clones). The isolated total DNA was analyzed by the E4 qPCR to determine the adenoviral copy number bound to the surface of the cells. The level of CD133 expression was determined by flow-cytometry, and shown below the graph (gray closed curve: unstained, open curve: anti-CD133 Ab) (*P < 0.05). (**B**) Binding ability of AdML-TYML in human colon cancer cell lines. The flow-cytometry data showing different level of CD133 expression in colon cancer cell lines (LoVo: CD133^+^, LS174T: CD133^-^). The results of binding assay were shown as E4 copy number per ng DNA (*P < 0.05). (**C**) The anti-CD133 antibody significantly inhibited the binding of AdML-TYML to the CD133-positive target cells. (*n* = 3, *P < 0.05).

To determine the specificity of the AdML-TYML for CD133-expressing cells, the binding assay was performed in the cells showing different level of CD133 expression. In 293-derived clones expressing different levels of CD133, AdML-TYML showed very strong binding to the cells exhibiting high expression of CD133 (Clone #9 and #12), and conferred moderate binding to polyclonal cells showing moderate CD133 expression (Figure 2A). On the other hand, its binding to both parental 293 and 293-EV, neither of which express CD133, was none to minimal. We further assessed AdML-TYML binding in colon cancer cell lines which express known levels of CD133. The virus accomplished high binding on CD133 strongly-expressing LoVo cells, while its binding to CD133 negative LS174T cells was as weak as the parental 293 cells (Figure 2B). Adenovirus with wild type Ad5 fiber (AdML-5WT) bound both cell lines, reflecting the CAR expression observed in these cell lines (Figure 2B, data not shown for control cell lines). These data indicated the differential binding of AdML-TYML virus in accordance with the level of CD133 expression on their surface. To further confirm the role of CD133 for AdML-TYML infection, we analyzed the effect of CD133 inhibition onto the binding of AdML-TYML by employing an anti-CD133 antibody. Pretreatment with the anti-CD133 antibody significantly inhibited the binding of AdML-TYML to CD133^+^ cells (293-CD133 and LoVo, Figure 2C). This observation confirmed that the binding of Ad-TYML was specific to the CD133 molecule.

### Replication and cytocidal activity of CD133-targeted adenovirus *in vitro*

**Figure 3:**
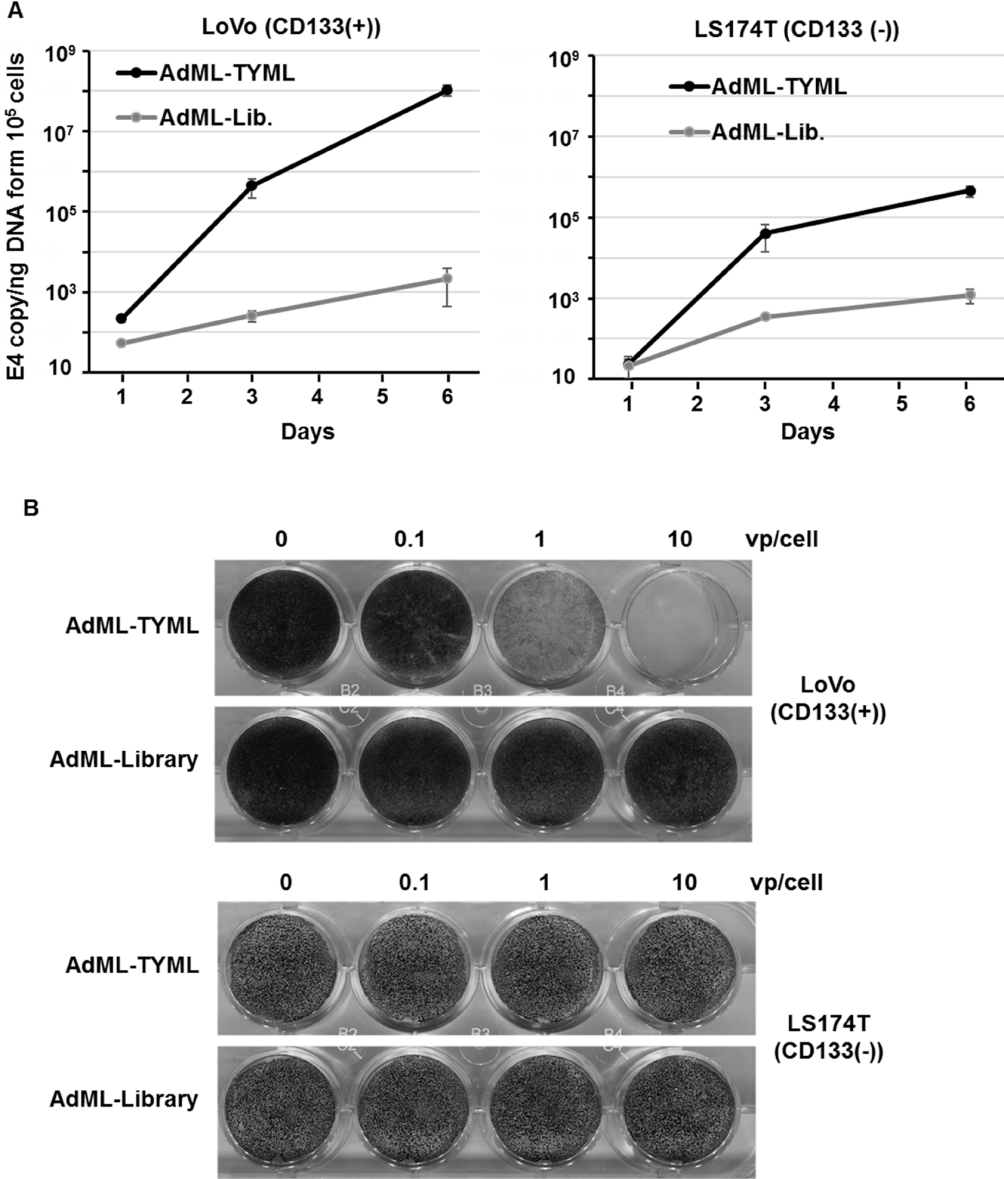
Viral replication and cytocidal activity of CD133-tergeted adenovirus *in vitro* (**A**) Selective replication of AdML-TYML in CD133 expressing colon cancer cells (LoVo). LoVo and LS174T cells were infected at 1 vp/cell with AdML-TYML or AdML-library, and viral copy number was measured by qPCR at day 3 and day 6. The result was shown as E4 copy number per ng DNA (*n* = 3). (**B**) Cytolytic effect of AdML-TYML *in vitro* in colon cancer cell line. LoVo and LS174T cells were infected at 0.1 to 10 vp/cell with AdML-TYML or AdML-library, with surviving cells stained by crystal violet. Staining was performed at day 6.

We next compared viral replication and cytolytic effect of CD133-targeted oncolytic adenovirus in CD133 positive or negative colon cancer cells. AdML-library before screening was used as a non-targeted negative control virus. AdML-TYML showed more than two order of magnitude higher amplification of viral DNA in the CD133^+^ cells (LoVo) compared to the CD133^−^ cells (LS174T) cells in a time-dependent manner (Figure 3A).

Non-targeted OAd which has random motif library on on the fiber knob (AdML-library) [25] did not replicate in both CD133^+^ and CD133^−^ cells (Figure 3A).

In the context of cytocidal effect, AdML-TYML showed strong cytolysis in CD133^+^ colon cancer cell line (LoVo) even at 1 vp/cell, but had no effect on CD133^–^ LS174T cells even at 10 vp/cell. The non-targeted OAd (AdML-library) showed no effect in either LoVo or LS174T cells (Figure 3B). The same correlation was observed in the 293 cell lines with or without CD133 overexpression (Supplementary Figure 1A). In 293-CD133 cells, the cytocidal effect of AdML-TYML was stronger than even AdML-5WT, which binds to CAR (Supplementary Figure 1B). These data indicated that AdML-TYML selectively replicated in and killed CD133-expressing cells.

### Effect of AdML-TYML on tumorigenic potential of cancer stem cells

**Figure 4:**
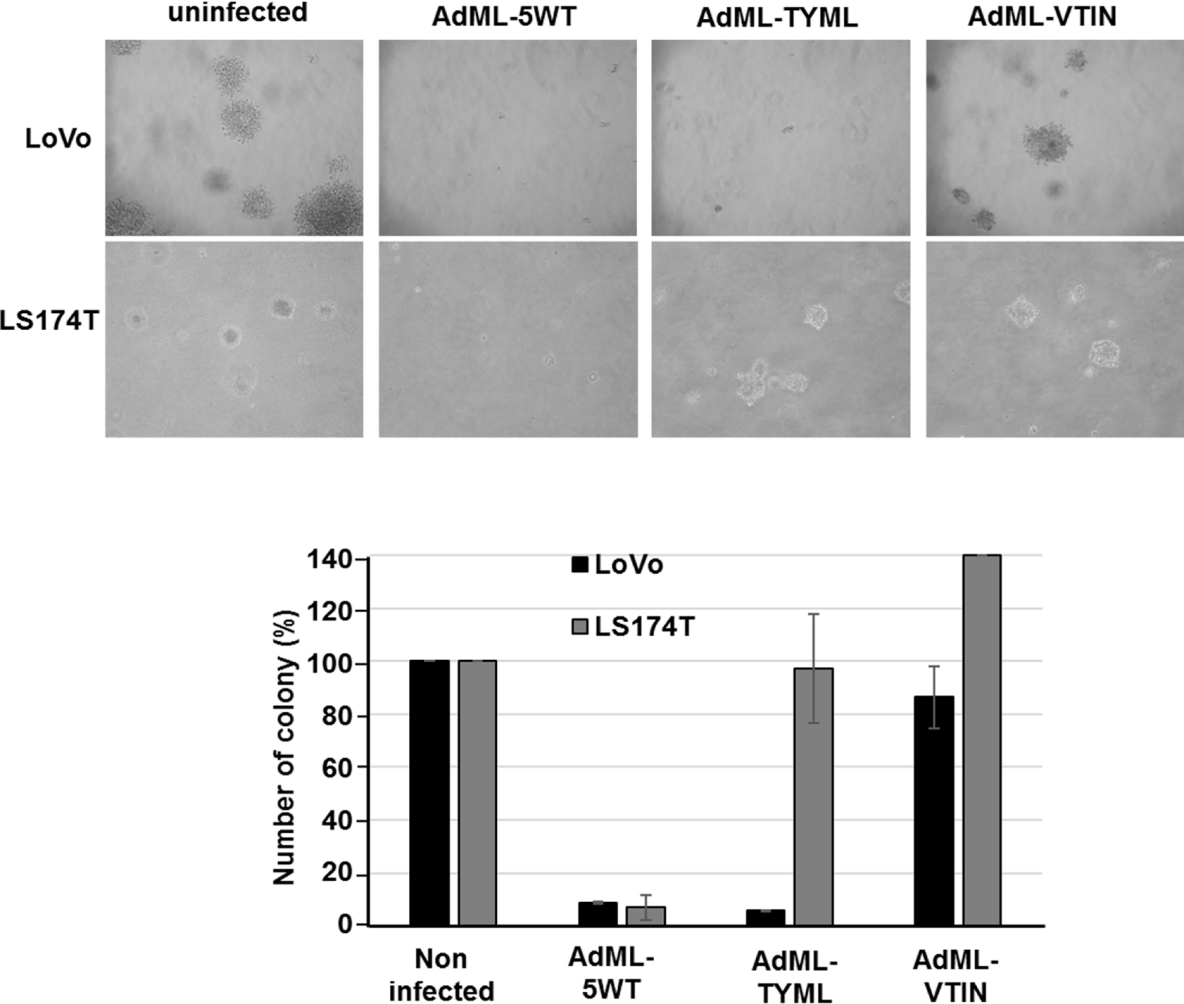
Effect of AdML-TYML in tumorigenic potential of Cancer Stem Cells Soft agar colony formation assay was performed in colon cancer cell lines in order to assess anchorage independent growth. LoVo (CD133^+^) and LS174T (CD133^-^) cells were infected with AdML-5WT, AdML-TYML (CD133-targeted OAd) and AdML-VTIN (mesothelin-targeted OAd) at 10vp/cell. Representative cell colonies in soft agar after 18 days are shown (Upper panels: LoVo cells, Lower panels: LS174T cells). The number of colonies is shown in the bar graph (*n* = 3).

CD133^+^ LoVo cells have been reported to be significantly more tumorigenic than CD133^–^ cells when assessed with clonogenic assays, including colony formation assay and mouse tumor establishment assay [26]. We first tested the effect of CD133-targeted Ad on LoVo cells using a soft-agar colony formation assay as part of the clonogenic assay. AdML-TYML eliminated colony formation in LoVo cells (CD133^+^), but not in LS174T cells (CD133^–^) (Figure 4). Another targeted OAd which selectively infects mesothelin expressing cells (AdML-VTIN) [25] did not suppress colony formation in either LoVo or LS174T cells at all. However, AdML-5WT, which binds to the CAR expressed by both LoVo and LS174T, eliminated colony formation in both cell lines (Figure 4). A simpler clonogenicity assay by colony formation without soft agar also showed the same trend, and AdML-TYML inhibited colony formation in LoVo cells. CD133-targeted OAd significantly suppressed clonogenic potential of the target cells (Supplementary Figure 2).

In order to assess the anti-cancer stem cell function of CD133-targeted AdML-TYML in a more stringent model, the mouse tumor establishment assay was performed. LoVo cells and LS174T cells were infected *in vitro* with AdML-TYML at 10vp/cell. After two hours of incubation, the cells were harvested and different numbers of cells (1 × 10^5^ or 1 × 10^4^ cells/site) were injected subcutaneously into the flanks of female athymic nude mice. Treatment with AdML-TYML resulted in significant reduction of the tumor formation in LoVo cells, but showed no effect on LS174T cells (Table 1). These data suggested that CD133-targeted OAd inhibited growth of cancer stem-like colon cancer cells in a manner dependent on CD133-expression of the target cells.

**Table 1:** Nude mouse tumorigenicity assay

Cell line	Day 7		Day14	
	Injected cell #		Injected cell #	
	1 × 10^5^	1 × 10^4^	1 × 10^5^	1 × 10^4^
LoVo	75% (3/4)	50% (2/4)	100% (4/4)	75% (3/4)
LoVo + AdML-TYML	0% (0/4)	0% (0/4)	0% (0/4)	0% (0/4)
LS174T	75% (3/4)	25% (1/4)	100% (4/4)	75% (3/4)
LS174T + AdML-TYML	25% (1/4)	25% (1/4)	75% (3/4)	50% (2/4)

### *In vivo* antitumor effect and virus replication of CD133-targeted adenovirus

**Figure 5:**
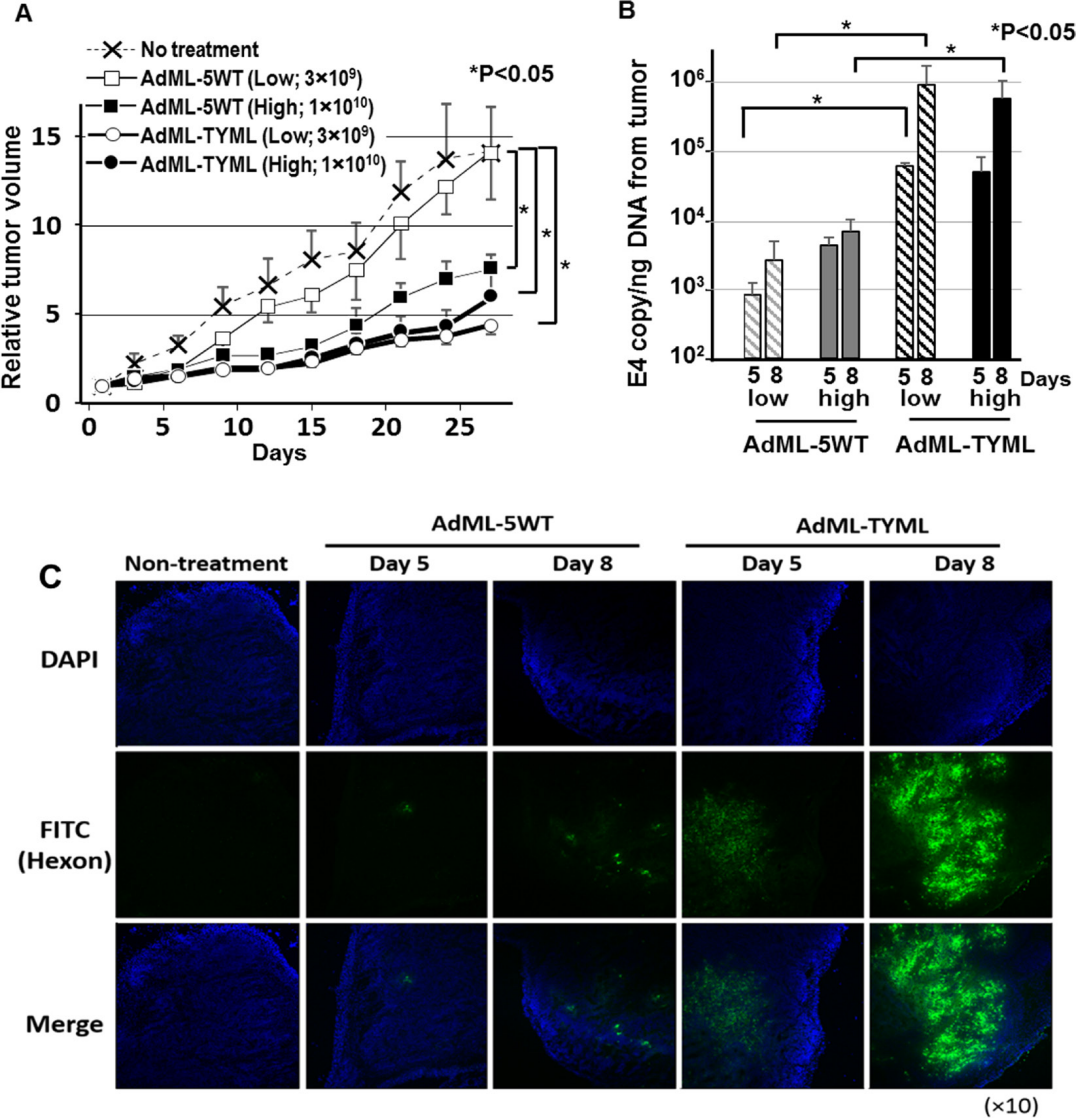
Antitumor effect and virus replication of CD133-targeted oncolytic adenovirus in established tumors (**A**) The anti-tumor effect of intratumorally-administered CD133-targeted OAd was analyzed in LoVo subcutaneous xenograft model. The control virus without targeting (AdML-5WT) or CD133-targeted virus (AdML-TYML) was *injected intratumorally* with different doses (1 × 10^10^ or 3 × 10^9^ vp/ tumor). Each symbol represents the mean relative tumor volume ± s.e.m. (*n* = 6) (*P < 0.05) (**B**) Viral copy number in the tumor specimens was analyzed by qPCR at day 5 and 8. The virus copy number as well as its increase between two time points was higher in AdML-TYML. The results are shown as E4 copy number per ng DNA (*P < 0.05). (**C**) At day 5 and 8, the expression of adenovirus late gene product (hexon) was assessed by immunostaining with the FITC-labeled anti-hexon polyclonal antibody (counterstained with DAPI). Staining and sections were performed in at least two independent experiments. Green; Ad hexon protein, Blue; nucleus (original magnification; ×100).

To assess the *in vivo* anti-tumor effect, CD133-targeted OAd (3 *×* 10^9^ or 1 *×* 10^10^ vp/tumor) was then injected intratumorally into xenografts established with LoVo cells when the tumors reached 5–7 mm diameter. The intratumoral (i.t.) administration of AdML-TYML exhibited significant antitumor effect, compared to AdML-5WT, which has wild type fiber (Figure 5A). Most interestingly, the injection of Ad-TYML at low dose (3 *×* 10^9^ vp) resulted in a remarkably greater anti-tumor effect than AdML-5WT at high dose (1 *×* 10^10^ vp), indicating significantly stronger anti-tumor effect of the CD133-targeted OAd. To investigate replication and intratumoral spread of the virus, we performed a separate experiment in LoVo tumors with the same setup, and the tumor samples at day 5 and 8 were assessed for viral copy number and viral structural protein (hexon) staining. The virus copy number on day 8 in tumors treated with AdML-TYML was more than two orders of magnitude higher compared to AdML-5WT (Figure 5B). Also, the copy number increase from day 5 to day 8 for AdML-TYML treated tumor was significantly higher than the increase of AdML-5WT for the same period. The staining of virus structural protein, hexon, in the tumor specimens at days5 and 8 after i.t. injection showed the vast majority of cells in the LoVo tumors treated with AdML-TYML expressed high levels of hexon protein, while treatment with non-targeted virus, AdML-5WT, resulted in much fewer hexon-expressing cells. Interestingly, immunostaining for the hexon protein showed remarkably more widespread distribution in the AdML-TYML treated tumors than the AdML-5WT treated tumors (Figure 5C). In summary, the CD133-targeted OAd showed stronger replication/spread and antitumor effect in CD133^+^ tumors *in vivo* compared to the non-targeted virus.

## DISCUSSION

In this study, we focused on CD133 as a target molecule of colorectal CSCs. CD133 overexpression has been associated with a poor therapeutic outcome and higher risk of metastasis in CRC patients [27, 28]. High-throughput screening of the high diversity targeting ligand library in OAd format on CD133 overexpressing cells resulted in the selective targeting ligand sequence, TYMLSRN. The insertion of this motif into AB-loop of the fiber/knob region of our oncolytic adenovirus resulted in a vector (AdML-TYML) which was able to bind and kill cells in a CD133-specific manner. These results show high versatility of our high-throughput screening system for generating infectivity-selective oncolytic adenovirus against a variety of targets as well as the potent and selective therapeutic potential of the virus equipped with the targeting motif resulted from this screening process.

The CD133-targeted AdML-TYML inhibited anchorage independent growth in soft agar colony formation assay, which is one of the features shared by many cancer stem cells/cancer initiating cells. In addition, more stringent inhibition of tumorigenicity was observed in nude mice tumor establishment assay (Table 1). Anti-CD133 OAd inhibits the cancer stem cell characteristics in two critical assays using cell lines where CD133 is a marker of cancer stem-like cells. These data indicate the potential value of our newly developed CD133 targeted vector for the prevention of tumor establishment or metastasis.

In addition to the effect on the cancer stem cell function during tumor establishment, strong anti-tumor effect was observed in established colon cancer xenografts in nude mouse model (Figure 5A). A very important observation in this experiment was the effect of CD133-targeted OAd, which is attacking the cancer stem-like cell population in the tumor, was evidently stronger than the OAd with wild type fiber that is expected to infect all cells within the tumor indiscriminately. When we observed the viral distribution by immunohistochemistry, the CD133-targeted virus showed far more spread in xenograft tumor compared to the wild type OAd (Figure 5C). Historically, distribution of intratumorally administered viral vectors is relatively limited to the tissue along the needle track, and therefore has issue of limited intratumoral spread [29]. Several reports have shown that connective tissue and extracellular matrix may have a prominent role in inhibiting viral spread [30, 31], and these non-cancer cells expressing CAR existing in the tumor may absorb the viruses and impairs the therapeutic potency. Our new vector, does not bind to CAR (Figure 2), and is not captured by CAR positive cells in the tumor. While wild type fiber OAd spread in tumors was reported to be restricted in clinical studies [32, 33], our data with AdML-TYML targeting CD133 is potentially more effective despite a smaller target population and shows a hope for better therapeutic effect through reduction of virus trapping by the non-cancer cells in the tumor.

Several therapeutic strategies targeting CD133 have been generated and have shown therapeutic effects for various types of cancer [34–38]. However, CD133 expression is likely to be oscillating, meaning that one cell showing CD133 expression at a given time point may become CD133^-^ at another time point, and vice versa [39]. As a result, oncolytic virus can be a more effective tool to eliminate a CD133^+^ cells than an anti-CD133 antibody because progeny of oncolytic virus is continuously produced in the tumor and keep attacking CD133^+^ cancer cells.

In the context of CD133-targeted oncolytic virus therapy, Bach et al. genetically engineered CD133-targeted measles virus which is equipped with a single-chain antibody (scFv) against CD133 [40]. They reported that this virus selectively eliminate CD133^+^ cells from tumor tissue [40]. These data suggest that the strategy of CSCs-targeted oncolytic virus can be used for cancer therapy. Although these previous studies used anti-CD133 antibody to target CD133^+^ CSCs, we were able to generate CD133-targeted adenovirus which does not rely on the use of antibody. A scFv independent approach provides two advantages. The first is bypassing the difficulty of generating a functional scFv. It is widely known that joining the variable heavy-chain domain and the light-chain domain (VH and VL) of the parent monoclonal antibody produces scFv [41]. However, the efficiency of such process is not high, and cloning functional variable genes is still a bottle neck for the scFv generation technology [42]. In this sense, avoidance of this undependable step is beneficial for the throughput of the whole process. Additionally, replacing native targeting module in the virus capsid sequence has much less interference than virus packaging and replication. Incorporation of scFv into virus virion (particularly for Ad virion) frequently interferes with virus assembly. In the context of the affinity of targeting motif, generally one targeting peptide alone is often not sufficient to achieve high affinity. However, the Ad knob contains three targeting peptides per fiber because of a trimeric structure of the fiber, and the virus binds to the target receptor with multiple fibers simultaneously [43]. Therefore, the binding ability of the Ad (e.g. CD133-targeted OAd) may have affinity comparable if not better than that of the scFv antibody. Although further experimentation will be needed for direct comparison, we believe that this novel scFv-independent targeting modality of OAd based on the library screening have distinctive benefits.

CD133 is not the only definitive CSCs marker in CRC. As an example, the cancer stem-like cell population of LS174T cells expresses the CD44 glycoform [44], and does not express CD133. Therefore, this cell does not respond to the CD133-targeted OAd as shown in this paper. This also explains the *in vivo* tumorigenic potential of LS174T cells, despite low CD133 expression. In such a case, we can also develop additional OAds targeting CSCs by focusing other cancer stem cell markers, such a CD44 or CD24. Recent data suggest that CSC population is phenotypically heterogeneous among tumor types and even within the same tumor subtype [45, 46]. The versatility of our screening technique permits the application of the same approach to the identification of the targeting motifs against other cancer stem cell markers. In fact, we have successfully identified a targeted adenovirus to mesothelin [25], a surface glycoprotein overexpressed in pancreatic cancer, ovarian cancer and malignant mesothelioma [47, 48]. Considering the necessity to develop multiple OAds targeting several different CSC markers, our adenovirus library screening system is suitable for the development of CSCs targeted oncolytic viruses in a variety of tumors.

In clinical situations, systemic treatment will be advantageous for the treatment of metastatic cancer, while many of past clinical trials have focused on intratumoral injection. When this virus is used for systemic treatment, one potential drawback of targeting CD133 is possible off-target effects on the hematopoietic stem cell and endothelial progenitors, both of which express CD133. To overcome this issue, we can combine the cancer targeted-OAd with tumor specific promoters, such as COX-2 [49] or CXCR4 [50] promoters. Annabi et al. showed that high grade glioma correlated with high expression of CD133 and cyclooxygenase (COX)-2 [51]. Furthermore, CD133^+^CXCR4^+^ cancer cells have a highly aggressive metastatic capacity compared to CD133^+^CXCR4^−^ cells [52]. Therefore, the development of a promoter (e.g. COX-2, CXCR4 promoters)-controlled CD133-targeted OAd will be a promising and practical direction toward safer systemic administration in the patients with disseminated diseases, although further investigation will be needed.

In this project, we generated a novel CD133-targeted OAd with newly developed high-throughput library screening method, and the resultant virus showed significant inhibition of CD133 positive cancer stem-like cells. Inhibition of tumorigenicity as well as therapeutic effect in established tumor was observed in CRC *in vivo* models. The results of this work demonstrated a proof of principle to generate cancer stem cell specific adenoviruses, and also laid the foundation for future development of CD133-targeted OAd suitable for treatment of variety of cancers whose CSCs marker is CD133.

## MATERIALS AND METHODS

### Cells and plasmids

Authenticated human embryonic kidney (HEK) 293 cells and human colon cancer cell lines (LoVo and LS174T cells) were purchased from ATCC, immediately prepared frozen cell stocks and stored in liquid nitrogen freezer. Cells were passaged for fewer than 6 months after resuscitation. Cell line authentication was performed by ATCC using short tandem repeat DNA profiles. All cell lines were routinely maintained in ATCC-recommended conditions. 293-CD133 cells overexpressing CD133 were established by transfection with CD133-expressing plasmid, pcDNA3.1-CD133 (CD133 cDNA cloned into pcDNA3.1, Invitrogen), and were maintained with G418 (600 μg/ml, Invitrogen). Cells were incubated in a 37°C and 5% CO2 environment under humidified conditions. Human cDNA CD133 expression plasmid (EX-Z0396-M02) and empty vector plasmid (EX-NEG-M02) were obtained from GeneCopoeia.

### Adenovirus design

The CD133-targeted adenovirus (AdML-TYML) has a TYMLSRN peptide motif in place of the primary CAR-binding domains in AB-loop of fiber knob, while mesothelin-targeted adenovirus (AdML-VTIN) has a VTINRSA peptide motif [25]. AdML-5WT, control adenovirus, has a wild-type AB-loop peptide sequence [25]. All viruses have a wild-type E1 gene, a single lox P site in the E3 gene as described previously [25]. The titer of the viruses was determined by optical absorbance at 260 nm and plaque assay [53].

### Adenovirus library with random peptide sequence in the AB-loop

Adenovirus-formatted fiber library was constructed with the rescue virus system as we previously reported [25]. This system enables high efficiency CRE-lox recombination between the shuttle plasmid coding Ad fiber library and the rescue virus. The rescue virus (AdMLΔF), generated with the shuttle plasmid pMLΔF, has a wild-type E1 gene, a single loxP site replacing the E3 gene, and a deletion of its fiber region (79.4–91.3 m.u.). The production of this virus was performed with fiber compensating cell line, 644 cells. The shuttle plasmids of the fiber library (pMLAB-lib) included a 76.1–100 map unit (m.u.) of the adenoviral genome with a single loxP site and library sequences in the AB-loop region of the fiber after a part of E3 region was deleted (79.4–84.8 m.u.)[54]. The 293CRE-69 cells (1 × 10^6^ cells in a 6cm culture dish) were infected with the rescue virus (AdMLΔF, 1 × 10^4^ vp/cell) for two hours. After 24 hours of incubation at 37^°^C, cells were transfected with 5 μg of the AB-loop shuttle plasmid (pMLAB-lib) containing seven amino acid random library and one loxP site at the immediate upstream of the fiber gene. The recombinant Ad or Ad-library was harvested 48 hours later. In the recovered virus, the viral DNA sequence coding seven amino acids including major CAR-binding domains was replaced with those coding a random seven amino acid library.

### Screening of CD133-targeted adenovirus

293-EV or 293-CD133 cells (5 × 10^7^ cells/10 cm dish) were infected with an Ad library at 100 vp/cell (5 × 10^9^ vp/10 cm dish). After 5–7 days following the infection, the viral solution was rescued. For each subsequent round of screening, a ten to twenty percent of the viral solution volume from the previous round was re-infected to the target cells, and the screening processes were repeated several times until observing convergence of library sequence. The DNAs were extracted from the viral solutions of each round, and the sequence of the AB-loop region was amplified by PCR with the following primers; AB-loop-S 5′-AAGCTAACTTTGTGGACCAC-3′ and AB-loop-AS 5′-ACTGCCACATTTTGTTAAGA-3′. The PCR product was purified with QIAquick PCR Purification Kit (Qiagen, Hilden, Germany) following the manufacture's instruction. The PCR products were then cloned with TOPO TA Cloning Kits for Sequencing (Invitrogen).

### Binding assay

One day after seeding (1 × 10^6^ cells/6 well plate or 1 × 10^5^ cells/12 well plate), the cells were infected with virus at 100 vp/cell. After two hours of incubation at 4^°^C, the cells were washed with PBS in order to prevent internalization of the virus into the cells, and the DNA was isolated. The viral infectivity was shown as E4 copy number per ng DNA as we described previously [55].

### Binding inhibition assay with Anti-CD133 antibody

The 293-CD133 cells were treated with the monoclonal anti-CD133 antibody (CD133/2 (293C3)-PE, Miltenyi Biotec) for 2 hours at a final concentration of 5μg/ml. After antibody treatment, the binding assay was performed as described above.

### Analysis of viral replication

The colon cancer cell lines (LoVo and LS174T) in 12-well plates (1 × 10^5^ cells per well) were infected with viruses (1 vp/cell). Then, the growth medium was harvested at 1, 3 and 6 d after infection to assess progeny production. The viral solution was treated with 0.1 U/μl of DNaseI at 37^°^C for 15 minutes for eliminating non-capsidated DNA. The DNA was purified with QIAamp DNA Mini Kit. The total viral copy number was analyzed with the E4 primers by SYBRGreen quantitative PCR (qPCR).

### Flow-cytometry

Cultured cells (≅2 × 10^5^) were dissociated with trypsin, washed, and resuspended in phosphate-buffered saline (PBS) plus FcR Blocking Reagent (Miltenyi Biotec Inc. # 130-059-901) then incubated with anti-CD133 phycoerythrin (PE)-conjugated antibody (clone 293C3-PE diluted 1:11, Miltenyi Biotec Inc.) for 0.5 hour at 4^°^C. Finally, cells were washed twice with PBS and analyzed with flow-cytometer (BD FACS CantoII: BD Biosciences, Franklin Lakes, NJ, USA).

### Analysis of cytocidal effect by crystal violet staining

One day after cells were plated (12-well plate, 1 × 10^5^ cells/well), the AdML-5WT or AdML-TYML virus was added at 0.1, 1, and 10 vp/cell. After 6 days of cultivation, the cells were fixed with 10% buffered formalin for 10 minutes, and stained with 1% crystal violet in 70% ethanol for 20 minutes, and then washed with water and dried.

### Soft agar colony formation assay

Colorectal cancer cells (1 × 10^5^ cells) were plated in 6-well plates then infected with AdML-TYML or control virus (AdML-5WT) at 10 vp/cell in 1ml of DMEM with 5% fetal bovine serum at 37°C for 2 hours. After incubation, the cells were washed and the medium was replaced with fresh growth medium. Two days after the infection, the cells were trypsinized and counted. Over the bottom layer (5 ml of the same medium containing 0.7% agar), 5 × 10^4^ infected LoVo cells were plated per 6-cm culture dish as a suspension in 3 ml of DMEM containing 10% fetal bovine serum and 0.35% agar. Plates were incubated at 37°C for 3–4 weeks until colonies were formed. Colonies were stained with 0.005% crystal violet. Total number of colonies was counted under a light microscope.

### Colony formation assay

CRC cells (1 × 10^5^ cells) was plated in 6-well plates then infected with AdML-TYML or control virus at 0.1 to 100 vp/cell in 1ml of DMEM with 5% fetal bovine serum at 37°C for 2 hours. After incubation, the cells were washed and the medium was replaced with fresh growth medium. At 2 days after the infection, the cells were trypsinized and counted. Five hundred treated cells were plated per 10-cm culture dish. Ten days later, cells were fixed in 4% PFA and stained with 0.5% crystal violet and colonies were manually counted.

### Nude mouse tumor establishment assay for CSCs

To analyze the tumor-initiating capacity of colon cancer cells after treatment with CD133 targeted versus, LoVo and LS174T cells were infected with AdML-TYML at 10 vp/cell. After 2 hour incubation, the cells were trypsinized and certain number cells (1 × 10^5^ or 1 × 10^4^ cells) were inoculated subcutaneously into the flank of the female nude mice. The mice were maintained under standard conditions according to the institutionally approved animal experimental protocol. Tumor appearance was inspected weekly by visual observation and palpation. Animal experiments were terminated one month after cell injection.

### *In vivo* therapeutic effect in established tumors

To analyze the anti-tumor effect in an *in vivo* model, 2 × 10^6^ of LoVo cells were inoculated subcutaneously into the flank of the female nude mice, and 3×10^9^ vp or 1 × 10^10^ vp/tumor of AdML-TYML virus or control AdML-5WT virus was intratumorally injected when the diameter reached 5–7 mm. The condition of the mice was monitored daily, and the tumor diameter was measured twice a week. The tumor volume was calculated as Width^2^ × Length/2. The animal experiments were performed in accordance with the institutionally approved animal experimental protocol.

In a separate experiment under same conditions, the mice were sacrificed at day 5 and 8. The DNA was purified from frozen tumor tissue by using QIAamp DNA Mini Kit, and the adenoviral DNA copy number of the E4 region was quantified by qPCR starting from 20ng DNA. The expression of adenoviral hexon protein in the tumor was analyzed by immunostaining [25, 53]. All slides were scanned at ×10 objective lens magnifications using a Nikon Eclipse TS100 microscope.

### Statistical analysis

Statistical analyses of *in vitro* and *in vivo* viral effects were carried out with Excel (Microsoft, Redmond, WA, USA). Statistical comparisons between two groups were evaluated by Student's *t*-test, and analysis of variance (ANOVA) including Dunnett's test was used for pair-wise comparisons between treated group and control group. *P* values less than 0.05 were considered statistically significant.

### SUPPLEMENTARY MATERIALS AND FIGURES



## Abbreviations

OAd: Oncolytic Adenovirus
CRC: Colorectal cancer
CSCs: Cancer Stem Cells
CAR: Coxackie Adenovirus Receptor
scFv: single-chain antibody
COX 2: singlecyclooxygenase 2
