# Supplementary material for: CD133-targeted oncolytic adenovirus demonstrates anti-tumor effect in colorectal cancer
Source: Oncotarget. 2017 Jun 2;8(44):76044–56. doi: 10.18632/oncotarget.18340 (PMC5652684; doi:10.18632/oncotarget.18340)
Supplement: Supplementary file 1 [file oncotarget-08-76044-s001.pdf]

CD133-targeted oncolytic adenovirus demonstrates anti-tumor effect in colorectal cancer

Supplementary Materials

MATERIALS AND METHODS

Generation of CD133-targeted OAd

CD133-targeted shuttle plasmid (pML-TYML). Three PCR products were prepared; The library PCR fragment was prepared from the synthesized oligonucleotide template (5′-AAGCTAACTTTGTGG ACCACACCAGCTCCATCTCCTAACACTTATATG CTGTCGCGTAATGATGCTAAACTCACTTTGGTCT TAACAAAATGTGGCAGT-3′) with the primers 5′-AAGCTAACTTTGTGGACCAC-3′ and 5′-ACTG CCACATTTTGTTAAGA-3′. For the upper franking region (709bp), Ad5 genome served as a template for PCR with the primers 5′-AATTGCTAGCCC TGCAAACATCAG-3′ (AB-upper-S) and 5′-GGTCC ACAAAGTTAGCTTATC-3′ (AB-upper-AS). For the lower franking region (442bp), fragment was prepared with the primers 5′-TTAACAAAATGTGGCAGTC AA-3′ (AB-lower-S) and 5′-AATTCAATTGAAAA ATAAACACGTTGAA-3′ (AB-lower-AS). These PCR products were mixed with an equal molar ratio and ligated by PCR reaction without primer. Subsequently, the final PCR was carried out with the primers AB-upper-S and AB-lower-AS. The PCR product digested with NheI and MunI was ligated into the same sites of pMLABΔSK.

The rescue virus (AdMLΔF), generated with the shuttle plasmid pMLΔF, has a wild-type E1 gene, a single loxP site replacing the E3 gene, and a deletion of its fiber region (79.4–91.3 m.u.). The production of this virus was performed with fiber compensating cell line, 644 cells. The shuttle plasmids of the CD133-targeted (pML-TYML) included a 76.1–100 map unit (m.u.) of the adenoviral genome with a single loxP site and TYMLSRN in the AB-loop region of the fiber after a part of E3 region was deleted (79.4–84.8 m.u.). The 293CRE-69 cells (1×10^6 cells in a 6 cm culture dish) were infected with the rescue virus (AdMLΔF, 1×10^4 vp/cell) for two hours. After 24 hours of incubation at 37°C, cells were transfected with 5 µg of the shuttle plasmid (pML-TYML). The recombinant Ad was harvested 48 hours later. In the recovered virus, the viral DNA sequence coding seven amino acids including major CAR-binding domains was replaced withCD133-targeted sequence (a.a. TYMLSRN).

Immunostaining

The presence of Ad particles in the tumor xenografts was assessed by immunofluorescence detection of the hexon protein. LoVo tumors were dissected, after fixation (buffered formaldehyde), the specimens were frozen and cut into 7-µm slices. The slices were embedded onto silane-coated slides and stained with FITC-conjugated anti-hexon goat polyclonal antibody (Millipore, AB1056F) and counterstained with DAPI.

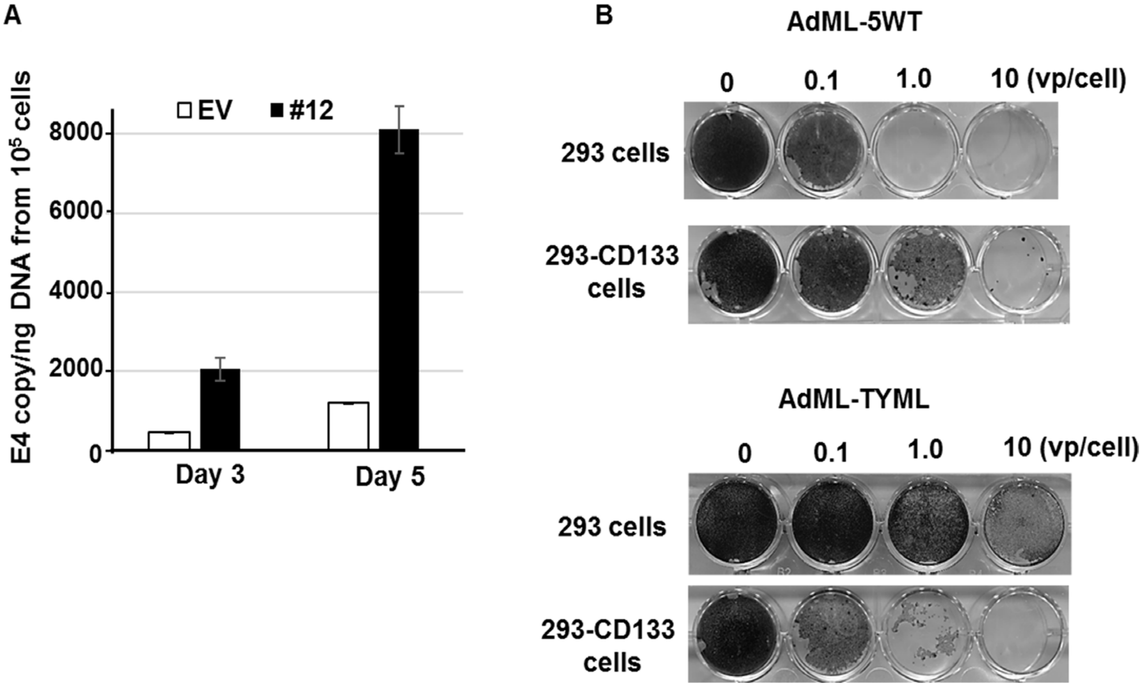

Supplementary Figure 1: Viral replication and cytocidal activity of CD133-tergeted adenovirus in CD133 overexpressing cells. (**A**) AdML-TYML showed very rapid amplification in CD133 expressing 293 cells (#12), but not in the cells transduced with empty vector (EV). (n = 3). (**B**) Cytolytic effect of AdML-TYML in vitro in 293 or 293-CD133 cells. Cells were infected at 0.1, 1.0, 10 vp/cell with AdML-TYML and AdML-5WT, with surviving cells stained by crystal violet. Staining was performed at day 5.

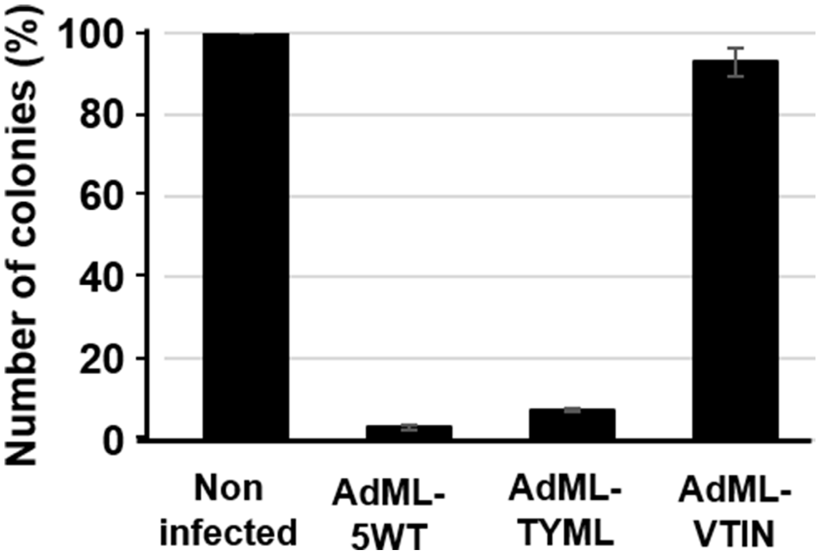

Supplementary Figure 2: Colony formation assay in LoVo cells. As compared to parental LoVo cells, LoVo-AdML-5WT and LoVo-AdML-TYML cells showed decreased colony numbers and after 7 days of culture on plates. Colony cells more than 50 cells were counted as one positive colony.
